# Synthesis of Magnetic Metal-Organic Frame Material and Its Application in Food Sample Preparation

**DOI:** 10.3390/foods9111610

**Published:** 2020-11-06

**Authors:** Jingying Yang, Yabin Wang, Mingfei Pan, Xiaoqian Xie, Kaixin Liu, Liping Hong, Shuo Wang

**Affiliations:** 1 State Key Laboratory of Food Nutrition and Safety, Tianjin University of Science and Technology, Tianjin 300457, China; yangjy0823@126.com (J.Y.); wyb1026585098@126.com (Y.W.); panmf2012@tust.edu.cn (M.P.); qianxx8135@163.com (X.X.); Liukx2019@163.com (K.L.); honglpstu@163.com (L.H.); 2 Key Laboratory of Food Nutrition and Safety, Ministry of Education of China, Tianjin University of Science and Technology, Tianjin 300457, China

**Keywords:** magnetic metal-organic frame material, food contaminants, sample preparation, synthesis and application

## Abstract

A variety of contaminants in food is an important aspect affecting food safety. Due to the presence of its trace amounts and the complexity of food matrix, it is very difficult to effectively separate and accurately detect them. The magnetic metal-organic framework (MMOF) composites with different structures and functions provide a new choice for the purification of food matrix and enrichment of trace targets, thus providing a new direction for the development of new technologies in food safety detection with high sensitivity and efficiency. The MOF materials composed of inorganic subunits and organic ligands have the advantages of regular pore structure, large specific surface area and good stability, which have been thoroughly studied in the pretreatment of complex food samples. MMOF materials combined different MOF materials with various magnetic nanoparticles, adding magnetic characteristics to the advantages of MOF materials, which are in terms of material selectivity, biocompatibility, easy operation and repeatability. Combined with solid phase extraction (SPE) technique, MMOF materials have been widely used in the food pretreatment. This article introduced the new preparation strategies of different MMOF materials, systematically summarizes their applications as SPE adsorbents in the pretreatment of food contaminants and analyzes and prospects their future application prospects and development directions.

## 1. Introduction

Food is the material basis for human survival, and food safety issues are a great concern of the international community. The existence of food contaminants such as residues of pesticide and veterinary drugs, toxins, illegal additives, heavy metal ions etc., is an important aspect causing the problem of food safety and has attracted wide attention from researchers [1,2,3]. Therefore, various strategies for the detection of contaminants in food have also been developed [4,5]. So far, the analysis strategies based on high-precision analytical instruments are still the main detection method of food pollutants because of the merits of high accuracy and sensitivity [6,7,8]. At the same time, some rapid detection methods and devices have been developed to realize convenient, rapid, low-cost and real-time analysis of food contaminants [9,10,11]. Because the food matrices are complex and contaminants are usually trace present, one of the key problems to limit the detection of food contaminants is sample pretreatment [12]. It is necessary not only to isolate analytes from the sample as thoroughly as possible, but also to enrich them before detection and quantification, while isolated analytes from the primary matrix remove the impact of interfering substances. Besides, foods belong to the fast-moving consumer product and have high requirement on detection speed or throughput. Therefore, while perfecting the detection technology, it is extremely urgent to develop accurate efficient and fast sample pretreatment techniques for food matrices [13,14]. An ideal food sample pretreatment technology not only requires high sensitivity, selectivity and reliability, but also has fast detection speed and convenient operation. This is a very critical step in the process of target analysis and one of the important research directions in the field of analytical chemistry [15].

After several years of development, the technology of solid-phase extraction (SPE) with simple operation, short pretreatment time, low consumption of organic solvents and high recovery of analyte has gradually become the most commonly used pretreatment technology for food complex matrices [16,17,18]. Varieties of SPE adsorbing materials can not only be customized according to the target substance, but also can match with different analysis equipment, which meets the detection requirements of high selectivity, high specificity and high throughput [19,20]. And many different nanomaterials have been studied and reported in the preparation of SPE adsorbent [21,22,23]. Among them, magnetic SPE, as a new type of pretreatment process, uses functionalized magnetic materials as adsorbents, and under the action of an external magnetic field, these magnetic adsorbents can be efficiently separated from the matrix samples [24,25,26]. The magnetic SPE (MSPE) procedure has a series of advantages such as simple operation, low solvent consumption, and high enrichment efficiency, which was widely used in the fields of food analysis, biomedicine and environmental monitoring [27,28,29]. Therefore, the synthesis and performance research of various MSPE materials has become a research hotspot in recent years [30,31].

Metal-organic framework (MOF) materials are a kind of crystal materials with three-dimensional network structure formed by self-assembly of inorganic subunits and organic ligands through coordination bonds. The inorganic subunits of MOFs usually include metal clusters, metal ions, or central chains [32,33,34]. The diversity and arrangement of metal elements and organic ligands can form different frameworks and pore structures, making the MOFs have different adsorption, optical and electromagnetic properties [35,36,37,38]. These characteristics have made MOFs materials widely concerned and used in the fields of sample pretreatment, chemical catalysis, drug delivery [39,40,41,42]. One of the research hotspots on MOFs is combining with various functional nano- and micro-particles to prepare highly porous nanocomposites [43,44,45]. These materials have the merits of nanomaterials while retaining the special properties and structure of MOF, thus expanding the application range of MOF materials. Among them, magnetic MOF (MMOFs) materials introduce magnetic nanoparticles (MNPs) to the surface or pores of MOFs, which not only maintains the characteristics of large specific surface area, strong adsorption capacity, chemical selectivity of MOFs, but also gives the material magnetic properties [46,47]. This kind of composite overcomes the shortcomings of MOFs which are easy to collapse under acid or alkaline conditions, thus increases the machinability and operability of MOFs, which has expanded the application range of MOFs in actual sample pretreatment [48,49]. As a new type of functional material, MMOFs materials have been widely used as SPE sorbent for the pretreatment of complex matrix, especially for the enrichment and separation of trace contaminants in food samples [50,51,52].

Therefore, this article summarizes the different preparation strategies of MMOFs currently used as adsorbents for food sample pretreatment, reviews the application of MMOFs in the detection and control of food contaminants, and looks forward to the future development direction and application prospects of MMOFs in the field of food analysis.

## 2. Synthesis Strategies of MMOFs Composite

MMOFs composite have become a burgeoning star in separation science [53,54]. The synthesis of MMOFs materials usually used MOFs as the precursors, and introduced or combined with MNPs (alloys, ferrites, metal oxides, etc.) by physical or chemical means. This not only maintain the advantages of MOFs (porous, highly modifiable and high specific surface area), but also endow them with magnetic separation characteristics. Furthermore, regular pore structure of MOFs is beneficial to mass transfer and diffusion and offer confinement effects to avoid aggregation of analytes. These properties make MMOFs very suitable for the pretreatment and purification of food matrices [55,56,57,58]. At present, various MMOFs composites with different properties have been synthesized using different strategies for food analysis [59,60,61] (Table 1).

### 2.1. Hybrid Preparation Method

**Figure 1:**
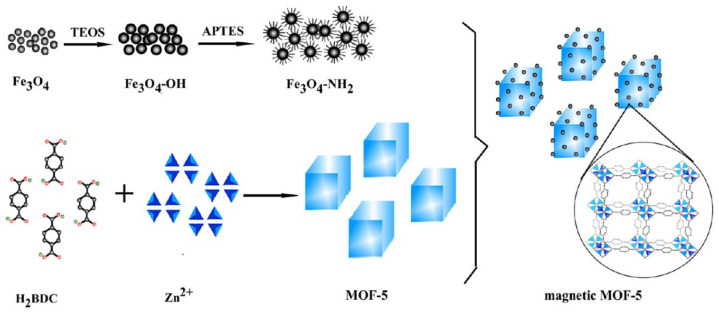
Schematic of the fabrication process of the hybrid magnetic MOF-5 using amino-functionalized Fe_3_O_4_ NPs [68]. Copyright Analytical Chemistry, 2013.

Hybrid preparation method is a process of directly mixing pre-prepared MNPs with MOFs, obtaining MMOFs composites through the ultrasonic or high-temperature polymerization procedures [62,63,64]. The MNPs can be modified on the surface of MOFs by electrostatic interaction or chemical bonding. The structure and performance of the prepared MMOFs can also be controlled by adjusting the interactions between MNPs and MOFs. The MNPs on the MOFs surface make the prepared MMOFs usually have better magnetic response [65,66]. Jiang et al., mixed cysteine functionalized Fe_3_O_4_ NPs with MIL-101(Fe) together under ultrasonication and obtained the Fe_3_O_4_/MIL-101(Fe) composite through electrostatic interaction [67]. It is worth noting that the Fe_3_O_4_/MIL-101(Fe) composite is more susceptible to magnetic fields and easier to isolate from aqueous solution, and exhibits much higher catalytic activity than the single component (Fe_3_O_4_ NPs and MIL-101) for the synthesis of 2,3-diaminophenazinedue to the synergistic peroxidase-like activity between the multiple active centers. A facile and efficient strategy about the synthesis of hybrid magnetic MOF-5 was illustrated in Figure 1 [68]. The pre-prepared amino-functionalized Fe_3_O_4_ NPs were introduced on the surface of MOF-5 via chemical bonding to improve the stability and structure uniformity of the hybrid microcrystals, which allows for facile withdrawal of the porous materials by magnetic decantation. Peña-Méndez et al., prepared one MMOFs Fe_3_O_4_@(Fe-(benzene-1,3,5-tricarboxylic acid) using the hybrid preparation method [69]. The obtained MMOFs was demonstrated to have high specific surface area (803.62 m^2^/g), good stability, strong magnetic response characteristics, which was further applied as a solid adsorbent of MSPE for food analysis.

Strictly speaking, this hybrid preparation method can prepare any required MMOFs with strong applicability. However, since the combination of MNPs and MOFs is not strong enough, causing easily separate from each other. Moreover, it is usually necessary to pre-prepare excessive amounts of MOFs during the preparation process, resulting in higher production costs [70].

### 2.2. In-Situ Growth Method

#### 2.2.1. In-Situ Growth of MOFs on MNPs

**Figure 2:**
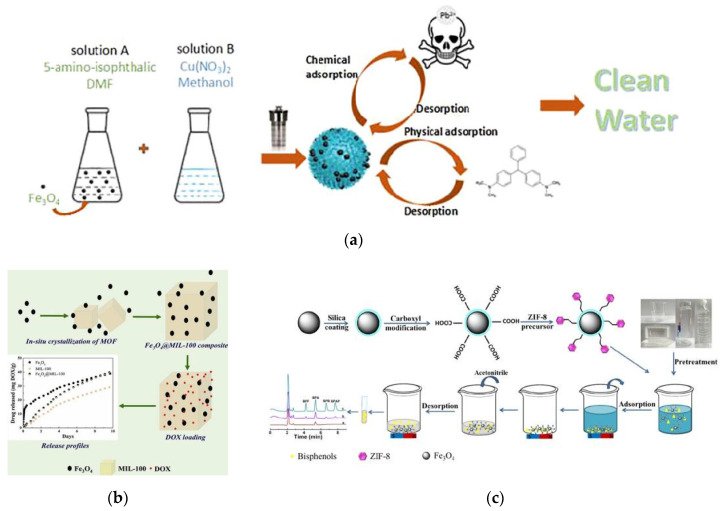
(**a**) Synthesis process of a Cu-MOFs/Fe_3_O_4_ composite by in-situ growth of Cu-MOFs doping with Fe_3_O_4_ NPs [73]. Copyright Colloids & Surfaces A, 2017. (**b**) In-situ synthesis of Fe_3_O_4_@MIL-100 doping with different amounts of Fe_3_O_4_ NPs [74]. Copyright Microporous & Mesoporous Materials, 2018. (**c**) In-situ growth of ZIF-8 on the surface of Fe_3_O_4_@SiO_2_ microspheres and its application [75]. Copyright New Journal of Chemistry, 2020.

In-situ growth of MOFs on MNPs, as its name suggests, the MOFs are grown in-situ on the surface of the MNPs added to the MOF precursor solution under ultrasonic or hydrothermal conditions. This method guarantees the structural integrity of the MMOFs to the greatest extent, so the obtained MMOFs materials have good adsorption performance [71,72]. Figure 2a has shown the synthesis process of a Cu-MOFs/Fe_3_O_4_ composite by in-situ growth of Cu-MOFs with doping Fe_3_O_4_ NPs. The adsorption capacities of the Cu-MOFs/Fe_3_O_4_ composite were found to be 113.67 mg/g for malachite green and 219.00 mg/g for Pb^2+^, respectively, which are significantly higher than other reported materials [73]. Different amounts of Fe_3_O_4_ NPs used would obviously affect the performance of in-situ synthesized MMOFs (Figure 2b) [74]. The obtained Fe_3_O_4_@MIL-100 composites were observed to have higher capacity in the application of loading the drug doxorubicin with the porous carriers. The highest loading capacity (about 19% mass) was observed for MIL-100 composites containing about 16% mass of Fe_3_O_4_ particles.

The in-situ growth of MOFs on MNPs is relatively simple, which can be completed in one step. This preparation process can be carried out at room temperature, which eliminates the need for complex carbonization processes at high temperatures, greatly reducing the preparation difficulty and shortening the preparation time, so it is very suitable for industrial production [76,77]. Figure 2c illustrated the in-situ growth of zeolitic imidazolate framework (ZIF)-8 on the surface of Fe_3_O_4_@SiO_2_ microspheres at room temperature and its application. This Fe_3_O_4_@SiO_2_/ZIF-8 material with large surface area and high super paramagnetism has great adsorption capacity to the analyte bisphenol A (a typical toxic chemical substance) [75].

However, in in-situ growth of MOF on MNPs, it is difficult to prevent the direct nucleation and growth of MOFs in solution, and part of the MNPs is embedded in the pores of the MOFs, resulting in a reduction in specific surface area [78,79]. Additionally, a large number of unreacted magnetic nanomaterials existing in the solution after reaction has made the subsequent separation steps cumbersome and require a large number of organic solvents [80].

#### 2.2.2. In-Situ Growth of MNPs on MOFs

In this method, the prepared MOFs are dispersed in a mixture containing reagents for preparing MNPs. The growth of MNPs is carried out at the presence of MOFs, and MNPs is embedded on the surface of MOFs. The free MNPs can be separated by centrifugation, and the MMOFs and MOFs are separated under an external magnetic field.

**Figure 3:**
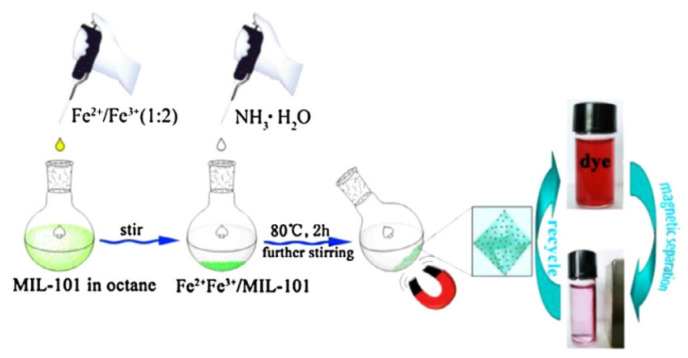
Schematic of the fabrication process of Fe_3_O_4_/MIL-101 [81]. Copyright Journal of the Taiwan Institute of Chemical Engineers, 2015.

Figure 3 shows the fabrication process of the magnetic hybrid Fe_3_O_4_/MIL-101 composite via in-situ growth of MNPs approach [81]. The solutions of MIL-101 and Fe^3+^/Fe^2+^ (2/1) were first mixed in n-octane, and then synthesized Fe_3_O_4_ NPs on MIL-101 crystal surface using ammonia solution as a precipitant. Characterizations of the synthesized Fe_3_O_4_/MIL-101 composite showed that the Fe_3_O_4_ NPs were uniformly coated on the outer-surface of MIL-101. This composite has a larger surface area, pore volume and higher positive charge than MIL-101, making it an excellent candidate for the adsorption and removal of anionic dyes from aqueous solutions.

The MMOFs prepared by in-situ growth of MNPs method have very more complete MOF structure, good adsorption property and magnetic response. However, there are some technical defects. A large number of free MNPs in the solution makes the subsequent separation steps complicated compared to other methods [82,83]. Before adding MNPs, the stability of MOFs should be strictly controlled to avoid collapse of MOFs skeleton. These problems need to be solved in the follow-up research.

### 2.3. Template-Directed Method

Template-directed method is an important strategy for preparing nanocomposite materials, and the most extensive method in the research of nanomaterials in recent years [84,85]. This method uses the materials with stable structure and controllable morphology as template and deposits the target material into the pores or surface of the template by physical or chemical methods. After removing the template, a nanocomposite material with standardized morphology and size of the template can be obtained [86,87,88]. The template-directed synthesis method can design the texture and structure of the template according to the morphology and performance requirements of the synthetic material to meet actual needs [89,90].

#### 2.3.1. Self-Sacrificial Template Method

**Figure 4:**
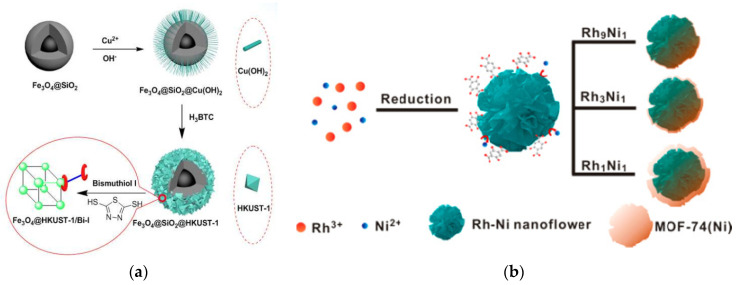
(**a**) Schematic diagram of the preparation of magnetic Fe_3_O_4_@SiO_2_@HKUST-1 composites [95]. Copyright Journal of Materials Chemistry A, 2015. (**b**) Schematic of the synthesis of Rh−Ni@MOF-74(Ni) composite [96]. Copyright ACS Applied Materials & Interfaces, 2016.

In self-sacrificial template method, MMOFs composites were prepared at room temperature by using metal oxide as metal source with MNPs pre-packaged in it [91,92]. The morphology and properties of the MMOFs can be controlled effectively [93,94]. Huang et al., have coated Fe_3_O_4_@SiO_2_ with Cu(OH)_2_ as the self-template, and converted Cu(OH)_2_ into HKUST-1 using ethanol and water as the solvent at room temperature (Figure 4a). The obtained MMOFs composite Fe_3_O_4_@SiO_2_@HKUST-1 was further applied as MSPE adsorbent to establish a sensitive, selective, simple-operated MSPE method for Hg^2+^ detection in water [95].

Bimetallic alloys NPs composed of noble-metals (Pt, Pd, and Rh) and transition metals (M = Fe, Co, Ni, Cu, and Zn) are low-cost and have superior performance in catalysis, which attracted increasing attention in recent years [97,98,99]. It is worth noting that the transition metal atoms in bimetallic NP tend to be oxidized and dissolved by dissolved oxygen, resulting in chemical dealloying in solution. Therefore, bimetallic NPs can be used as the self-sacrificial template to synthesis the required MNPs@MOF composite material with definite structure [100,101]. Chen and his co-workers reported a MOF-74(Ni)-encapsulated Rh-Ni hierarchical hetero structures (Rh-Ni@MOF-74(Ni)) using magnetic Rh-Ni-alloyed nanoflowers (NFs) as a self-sacrificial template [96] (Figure 4b). The encapsulation state and thickness of the formed MOFs shell were well-tuned via template dealloying by changing the Ni content in the Rh-Ni NFs template. More interestingly, when using Rh-Ni@MOF-74 (Ni) as the catalyst, due to the confinement effect of the MOF shell, enhanced catalytic performance was observed for the selective hydrogenation of alkynes to cis products.

#### 2.3.2. Emulsion Template Method

**Figure 5:**
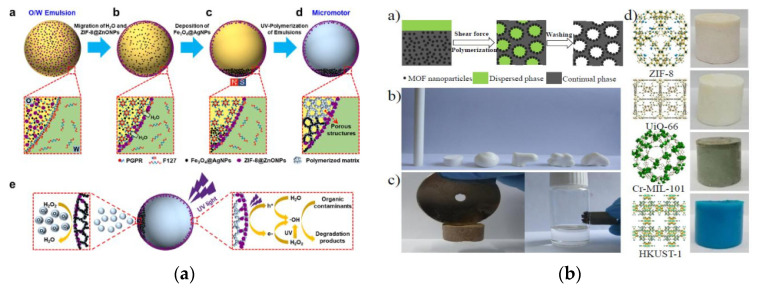
(**a**) Schematic illustration of the fabrication process of MOF-integrated photocatalytic micromotors [106]. Copyright ACS Applied Materials Interfaces, 2020. (**b**) Illustration of the interfacial assembly/polymerization fabrication procedure for the preparation of MMOFs [107]. Copyright Communication, 2013.

Emulsion template method is a new method of preparing porous materials in recent years [102,103]. This method generally uses emulsion microdroplets from microfluidics as template, and the target material is deposited on the surface of template by physical or chemical methods. After formation, the template was removed, leaving the neat pore structure [104,105]. Figure 5a showed a simple and general strategy for continuous fabrication of MMOFs [106]. Using Fe_3_O_4_@AgNPs integrated into controllable emulsion micro-droplets as template, uniform porous MMOFs decorated with Fe_3_O_4_@AgNPs at the bottom and ZIF-8@ZnONPs on the surface was successfully synthesized. Through the directional migration of emulsion droplets in the limited microspace driven by interface energy and magnetic field, the spatial position of ZIF-8@ZnONPs and Fe_3_O_4_@AgNPs in MMOFs can be precisely controlled. Different kinds of metal-based composite MOFs materials can grow in the polymer macropores to generate MMOF materials. Jin and his co-workers used emulsion droplet as template to prepare MMOF composite materials through interfacial nanoassembly/emulsion polymerization [107]. The fluid properties of the emulsion droplets can process the MOFs composite material into a specific task shape. The embedded Fe_3_O_4_ NPs offered the MOF composite magnetic properties and excellent mechanical stability (Figure 5b). Chen et al. prepared a magnetic porous polyacrylamide polymer using Fe_3_O_4_ and polyvinyl alcohol under water bath conditions, and successfully induced UiO-66 and Fe-MIL-101(-NH_2_) crystals of MOFs in the high-temperature emulsion template [108].

In emulsion template method, it can effectively avoid the decrease of specific surface area caused by the addition of MNPs, and can also construct macroporous polymers, thus accelerating the effective transport of matrix in the pore tunnel. However, this method requires a large amount of inorganic solvent and the preparation process is cumbersome [109].

### 2.4. Layer by Layer Self-Assembly Method

As a simple and efficient surface modification method, layer by layer (LbL) self-assembly technology has attracted increasing attention in the preparation of nanomaterials and promoted the development of material preparation research [110,111]. In the process of MMOFs preparation, before mixing with MOFs ligand solution, appropriate functional groups can be modified on the surface of magnetic nanomaterials, so that the MOFs ligands can gradually extend on the surface of magnetic nanomaterials and spontaneously assemble into MMOFs [112,113]. Generally, the chemical groups modified on the surface of magnetic nanomaterials can promote the growth of MOFs crystal nucleus, which is beneficial to the formation of core-shell structure, improving the stability of MMOFs [114,115].

**Figure 6:**
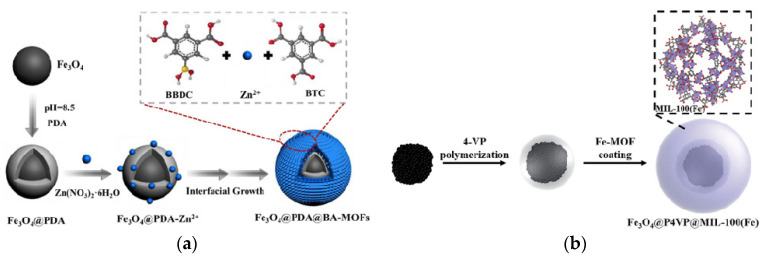
(**a**) Illustration of LbL assembly strategy to fabricate the Fe_3_O_4_@PDA@BA-MOFs composite [116]. Copyright Chemical Engineering Journal, 2018. (**b**) Schematic illustration of the synthesis of Fe_3_O_4_@P4VP@MIL-100(Fe) composite [118]. Copyright RSC Advances, 2017.

Figure 6a illustrated a LbL self-assembly strategy to fabricate Fe_3_O_4_@polydopamine (PDA)@boric-acid (BA)-functional MOFs (Fe_3_O_4_@PDA@BA-MOFs) for highly selective capture and separation of natural flavone from complex extraction samples [116]. The magnetic core Fe_3_O_4_ was prepared using a solvothermal reaction and self-polymerized of PDA onto the surface. Due to the hydroxyl and amino groups of PDA, Zn^2+^ was easily adhered to the core surface, which can promote the growth of MOFs crystal nucleus. The Fe_3_O_4_@PDA@BA-MOFs with ultrahigh surface area, uniform morphology, excellent hydrophilicity and strong magnetic responsiveness were demonstrated to have high selectivity, obvious binding kinetics, large adsorption capacity, and excellent reusability (above 91.23% at least six repeated adsorption-desorption cycles), which would be good candidates for the enrichment and purification of luteolin. Liu et al., prepared the magnetic composite of MOF-Fe_3_O_4_@HKUST-1/MIL-100(Fe) by LbL self-assembly method and applied it for the detection of methylene blue in foods [117]. Great recoveries in the range of 70–110% signified the application prospect of MMOFs as SPE adsorbent in the pretreatment of food matrices. Miao and the co-workers have prepared the core of Fe_3_O_4_@poly(4-vinylpyridine) (P4VP) and introduced the MIL-100(Fe) shell through LbL self-assembly [118] (Figure 6b). This novel magnetic Fe_3_O_4_@P4VP@MIL-100(Fe) composite with core–shell material was applied as the catalyst for selective oxidation of alcohols with remarkable reusability (maintained after ten reuses).

LbL self-assembly method can also precisely control the thickness or properties of MMOFs by adjusting the number of self-assembly cycles, and the reaction conditions are relatively mild. However, it usually takes a long time to obtain the desired thickness of MMOFs, and the types of self-assembled MOFs ligands are few, which limits the development of new MOFs with crystal structure.

### 2.5. Other Strategies for MMOFs Synthesis

**Figure 7:**
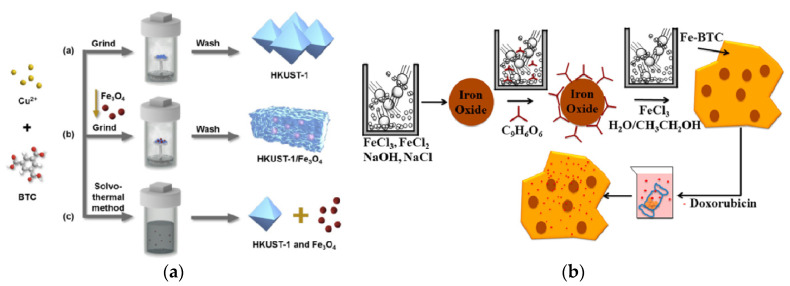
(**a**) Schematic diagram for MMOFs synthesis by the dry gel conversion method [121]. Copyright Journal of Hazardous Materials, 2017. (**b**) Schematic diagram for MMOFs synthesis by mechanical grinding method [124]. Copyright Inorganic Chemistry, 2018.

Dry gel conversion method was also used for the synthesis of MMOFs. In dry gel conversion method, the reactants were first separated from the solvent and steamed at a high temperature to induce the MOFs material to grow around MNPs [119,120]. The preparation of MMOFs by dry gel conversion method was first proposed by Tan et al., in 2017 [121]. Figure 7a has shown a synthesis process of MMOFs by dry gel conversion method. The solid separated from the solvent was stewing with *N*,*N*-dimethylformamide at high temperature to induce the growth of MOFs around MNPs, and successfully prepared a MMOFs material Fe_3_O_4_/HKUST-1. The characteristic of growing around the MNPs avoids the blockage of material pores caused by the presence of unreacted magnetic nanomaterials in the prepared MOFs material channel, reduces the loss of organic solvents to a certain extent, and needs less time for preparation. It’s worth noting that the uniformity of the MMOFs prepared by this method needs to be improved [122,123].

At room temperature, MOFs can be connected to the surface of MNPs with organic linker, which can promote the growth of MOFs. Through liquid-assisted grinding in water or ethanol, the MMOFs composite can be obtained [125,126,127]. This procedure for MMOFs synthesis is named “mechanical grinding method”. Bellusci et al., first verified the feasibility of the grinding method to obtain a magnetic composite of MOFs (Figure 7b). A magnetic Fe_3_O_4_ NPs were first prepared using ball grinding method by inducing the mechanical and chemical reaction, and further functionalized by neat grinding with benzene-1,3,5-tricarboxylic acid to obtain a composite consisting of Fe_3_O_4_ NPs encapsulated in a MOF matrix. The prepared MMOFs exhibits magnetic characteristics and high porosity and can be used as the carrier for targeted drug delivery and release [124].

Although there are only few types of MOFs ligands available and the properties of the prepared MMOFs are not uniform, this mechanical grinding method also has great potential in future studies on the preparation of MMOFs due to its simple synthesis process and low cost.

## 3. Applications of Magnetic MOFs for Food Contaminants Extraction

### 3.1. Residues of Pesticide and Veterinary Drugs

The residue of pesticide and veterinary drugs in food are the main contaminants in food, which has aroused wide concern [128,129,130]. In the process of crop production, pesticides such as insecticides and herbicides are used in large quantities and enter human and animal bodies through food intake, resulting in accumulative toxicity. For the production of animal-derived foods (meat, eggs, milk, etc.), various antibiotics and other veterinary drugs are used to treat livestock diseases and promote animal growth. Improper use of veterinary drugs resulted in their residues in food samples [131,132]. After the foods containing the residue of pesticides or veterinary drugs are ingested by human body, it may induce various allergic reactions or diseases, which bring great threat to human health.

**Figure 8:**
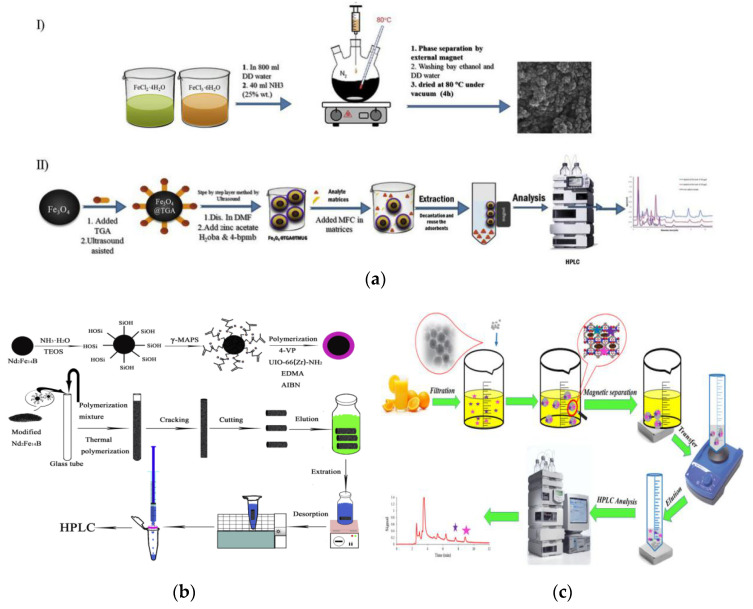
(**a**) Synthesis of Fe_3_O_4_@TGA@TMU-6 for MSPE of OPPs in water and rice [137]. Copyright Talanta, 2020. (**b**) Preparation and stir-bar sorptive extraction scheme of the hybrid UIO-66(Zr)-NH_2_ magnetic stir bar [139]. Copyright Microchemical Journal, 2018. (**c**) MSPE using Fe_3_O_4_@TMU-21 and HPLC analysis procedure of trace pyrethroids [140]. Copyright Microchemical Journal, 2019.

In the detection process of the residue of pesticide and veterinary drugs in food, MMOFs are characterized by easy preparation, simple operation and high adsorption efficiency, which can effectively reduce matrix interference and improve the detection sensitivity of the target [133,134,135,136]. Figure 8a has shown the synthesis of a MMOF (Fe_3_O_4_@ thioglycolic acid (TGA) @TMU-6) and the use for MSPE of some organophosphorus pesticides (OPPs) in rice and environmental water samples [137]. Due to the large surface area and unique porous structure, as well as the π-π, hydrophobic interaction between the analytes and MOF ligands, the prepared MMOFs composites have a high affinity for OPPs. The detection limit (LOD) of phosalone, chlorpyrifos, and profenofos achieved to 0.5, 1 and 0.5 μg/L, respectively. The Zn/Co-MOFs-derived magnetic nanoporous carbons synthesized by Li et al., via one-step carbonization also exhibit high specific surface area and high extraction efficiency for OPPs. Coupled with gas chromatography-flame photometric detection (GC-FPD), a simple, rapid, selective and highly sensitive analysis for trace five OPPs in fruits was achieved [138]. A UIO-66(Zr)-NH_2_ hybrid magnetic stir bar developed by Yang and the co-workers was successfully applied for adsorption and extraction of five sulfonylurea herbicides in food samples [139] (Figure 8b), which obtained higher extraction efficiency than the blank stir bar without UIO-66(Zr)-NH_2_. The LOD for five analytes was in the range of 0.04–0.84 μg/L with the recoveries of 68.8–98.1% in real samples. The UIO-66(Zr)-NH_2_ hybrid magnetic stir bar possessed excellent advantages of both supermagnetic performance and large specific surface area, which is an optimal adsorbent for food analysis. The MMOFs composites (Fe_3_O_4_@TMU-21) with a shell-core structure prepared by Yamini et al. through hybrid preparation method showed good selectivity and high recovery for pyrethrin pesticides in orange juice [140] (Figure 8c).

Up to now, more and more studies on the development and application of MMOFs for analyzing pesticide and veterinary drug residues were reported. The detection targets also extended to fluoroquinolones [141,142], sulfonamides [143], ampicillin [144], nitroimidazoles [145], benzoylurea insecticides [146], tetracycline [147], triazine herbicides [148] et al., Duo et al., synthesized a novel, spinel-like magnetic metal-organic skeleton material (Fe_3_O_4_-NH_2_@MOF-235) by a two-step solvothermal method, and used as SPE adsorbent for simultaneous extraction and determination of five benzoyl urea pesticides in honey, fruit juice and tap water samples [149]. The deposition and hydrophobic interaction between Fe_3_O_4_-NH_2_@MOF-235 and the analyte plays an important role in the adsorption process, and the maximum adsorption capacity can be reached within 5 min. The magnetic Fe_3_O_4_-NH_2_@MOF-235 composite combined with SPE-HPLC is demonstrated to be a convenient method for the detection of benzourea insecticides in actual samples.

### 3.2. Toxins

#### 3.2.1. Mycotoxins

Mycotoxin is a kind of secondary metabolites produced by fungi, which is the common contaminants in grains, oils and other foods [150,151]. Each step in the food production including processing, storage and transportation is susceptible to fungal infection, which is why mycotoxin pollution is difficult to avoid. Ingesting foods with high levels of mycotoxin can cause serious harm to human body [152,153]. The food matrix composition is complex and the content of mycotoxins in food is very low, which brings difficulties to the detection of mycotoxins contamination. MSPE technique using MMOFs as the purification material has high enrichment efficiency, which has been widely used in the analysis of mycotoxins in foods.

**Figure 9:**
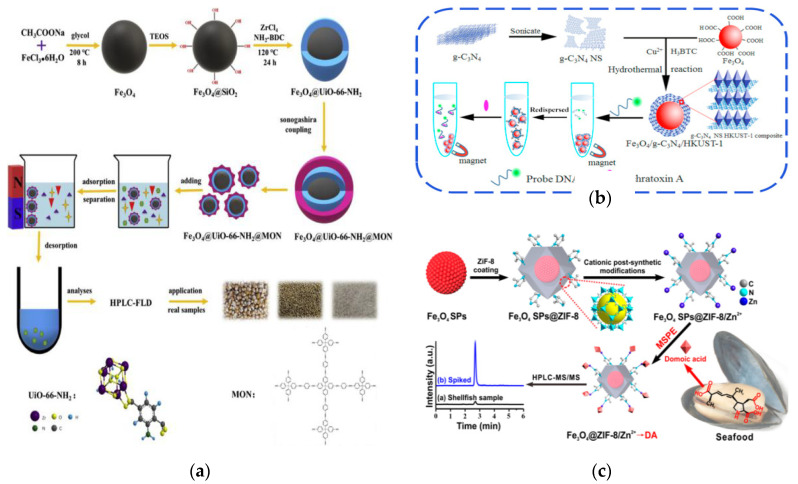
(**a**) Schematic illustration of the synthesis and application of Fe_3_O_4_@UiO-66-NH_2_@MON [154]. Copyright Journal of Hazardous Materials, 2020. (**b**) Biosensing detection of ochratoxin A based on Fe_3_O_4_/g-C3N4/HKUST-1 composite [157]. Copyright Biosensors and Bioelectronics, 2016. (**c**) Scheme for preparing Fe_3_O_4_ SPs@ZIF-8/Zn^2+^ particles and the use in MSPE for domoic acid detection [158]. Copyright Analytical Chemistry, 2019.

Aflatoxins, produced by filamentous fungal species, are the most toxic type of mycotoxins and great effort has been made in developing of adsorptive materials for effective probing the target aflatoxins. Li et al., have constructed a core-shell structure Fe_3_O_4_@UiO-66-NH_2_@ microporous organic network (MON) composite and used it as sorbent to separate aflatoxins [154] (Figure 9a). The obtained sorbent possessed excellent selectivity and sensitivity with LODs in the range of 0.15–0.87 µg/L by combination with HPLC analysis. And due to the presence of MON coating, the hydro-stability and adsorption efficiency of adsorbents were significantly improved. Another composite of MIL53(Al)-SiO_2_@Fe_3_O_4_, prepared in a typical Stöber synthesis process and ultrasonic agitation co-precipitation method, was applied in multi-component adsorption for aflatoxin B1 in winter herbal tea, which obtained a wider linear range (0.5–150 ng/mL), a lower LOD (0.5 ng/mL), and an acceptable recovery rate (70.7–96.5%) [155]. A MIL-101(Cr)/Fe_3_O_4_@SiO_2_@propylthiouracil composite developed by Sabeghi et al., was used to simultaneously isolate and determine four aflatoxins (B1, B2, G1, G2) in pistachio samples [156]. The developed strategy was shown to be facile, sensitive and accurate for aflatoxins detection, having great prospect for aflatoxins detection from other complex samples. Hu et al., prepared a MMOFs (Fe_3_O_4_/graphitic-phase carbon nitride(g-C_3_N_4_)/HKUST-1) through the LbL self-assembly method, which was used as a MSPE adsorbent to detect ochratoxin A in corn [157] (Figure 9b). Under the optimal conditions, the Fe_3_O_4_/g-C_3_N_4_/HKUST-1 exhibit better sensitivity (LOD: 2.57 ng/mL) and higher recovery (96.5–101.4%) for ochratoxin A, which confirmed the application prospect of magnetic MOFs as SPE adsorbent in food sample pretreatment.

#### 3.2.2. Algal Toxins

Due to climate change and large amounts of industrial wastewater discharge, environmental issues such as eutrophication of water and cyanobacteria blooms have also attracted widespread attention. Harmful algae can produce a large number of algal toxins to pollute water sources, which also can enter human body through the digestive tract, causing diarrhea, nerve paralysis, liver damage, and even death in serious cases [159,160].

Microcystin-LR with high content and strong toxicity, belonging to class 2B carcinogen, was released by cyanobacteria in eutrophic water. Chen et al., successfully synthesized Fe_3_O_4_@poly-dopamine(PDA)@Cu-MOFs and further applied to analyze traces of microcystin-LR [161]. It is found that the Fe_3_O_4_@PDA@Cu-MOFs composite material exhibits ultra-high surface area, strong magnetic response and excellent hydrophilicity. Combined with matrix-assisted laser desorption/ionization time-of-flight mass spectrometry (MALDI-TOF-MS), microcystin-LR in water was accurately and sensitively detected. Huang et al., fabricated the MMOFs (Fe_3_O_4_ superparticles (SPs) @ZIF-8/Zn^2+^) for the adsorption of domoic acid from food samples [158]. Through the strong electrostatic interactions and chelation with coordinatively unsaturated Zn^2+^ sites on the surface of magnetic MOFs, the adsorption of domoic acid can be finished within 5 min. By employing Fe_3_O_4_ SPs@ZIF-8/Zn^2+^ as sorbent for MSPE, followed by liquid chromatography-mass spectrometry (LC-MS), a facile, rapid, efficient, and sensitive detection of trace domoic acid in marine products was realized (Figure 9c).

### 3.3. Illegal Additives

**Figure 10:**
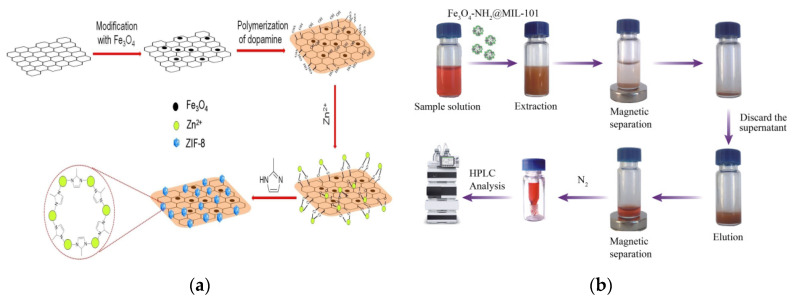
(**a**) Scheme of synthetic process of magG@PDA@ZIF-8 [167]. Copyright Chemistry Select, 2018. (**b**) MSPE for Sudan dyes using Fe_3_O_4_-NH_2_@MIL-101 as adsorbent [169]. Copyright Journal of Chromatography B, 2018.

As an important part of food industry, food additives play an irreplaceable role in food production, processing and storage. However, the emergence of illegal additives such as melamine, Sudan dyes and plasticizer compounds has brought new threats to food safety [162,163]. In the analysis of illegal additives in foods, MMOFs materials with simple preparation and high adsorption efficiency are also used for food matrix purification and trace contaminants enrichment [164,165,166]. Lu et al. have proposed a location-controlled strategy for functionalization of hydrophilic magnetic graphene by ZIF-8 MOFs to construct uniform and robust nanosheets (magG@PDA@ZIF-8) [167] (Figure 10a). The prepared magG@PDA@ZIF-8 has regular porous structure and large surface area with unique π-π stacking electron system, strong magnetism and excellent water dispersibility, which was further used to enrich nine phthalates in cola beverages. Yamini et al., synthesized the Fe_3_O_4_@TMU-24 materials were demonstrated an effective sorbent for preconcentration of the plasticizer compounds [168]. Shi et al., fabricated a MMOFs (Fe_3_O_4_-NH_2_@MIL-101) through in-situ synthesis method, which can be used for MSPE pretreatment to effectively isolate six Sudan dyes [169]. Sudan dyes were efficiently separated from the complex matrices using only 3 mg of Fe_3_O_4_-NH_2_@MIL-101 within 2 min, making pretreatment easier, faster and more effective. Figure 10b has shown the MSPE process for Sudan dyes detection using Fe_3_O_4_-NH_2_@MIL-101 as adsorbent. Zhou prepared a Fe_3_O_4_@PEI-MOF-5 material by a facile two-step solvothermal approach for effective enrichment of malachite green (MG) and crystal violet (CV) in fish samples [170]. Th new material was demonstrated to have high magnetization and chemical stability, a large surface area and good adsorption to MG and CV. By combining the Fe_3_O_4_@PEI-MOF-5 material with HPLC-MS, an effective enrichment and detection method for MG and CV was subsequently developed with linearity range of 1–500 ng/mL (MG) and 0.25–500 ng/mL (CV), LOD of 0.30 ng/mL (MG) and 0.08 ng/mL (CV), respectively.

### 3.4. Heavy Metal Ions

The analysis of heavy metal elements in food is another research hotspot of food contaminant detection [171,172]. The excessive discharge of industrial sewage containing heavy metals, the unreasonable use of pesticides containing heavy metals and other factors have resulted in the excessive heavy metals in the water and soil where the food raw materials are produced [173,174]. The pollution of heavy metals is difficult to be biodegraded and can enter human body through water or food chain, resulting in accumulative toxicity. Modern medicine research has proved that the accumulation of heavy metals is likely to induce cardiovascular, liver and kidney diseases and even cancers, which has posed a serious threat to human health [175]. The detection of trace heavy metals in food matrix also requires matrix pretreatment, target ion enrichment and other processes. The composite of MMOFs is simple to prepare, stable in structure and function, and can replace the traditional adsorbent to achieve more efficient and faster detection [176,177]. As a result, various MMOFs were synthesized and applied in the extraction of Hg^2+^ [178,179,180], Sn^2+^ [181], Cr^3+^ [182], Pb^2+^ [183] from food samples.

**Figure 11:**
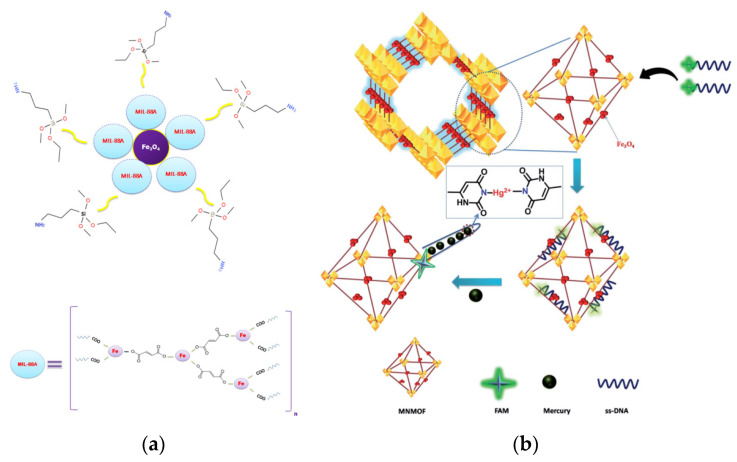
(**a**) Proposed structure of nFe_3_O_4_@MIL-88A(Fe)/APTMS [184]. Copyright Journal of Hazardous Materials, 2019. (**b**) Schematic illustration of the DNA fluorescence assay with MMOFs as a sensing platform [186]. Copyright RSC Advances, 2020.

Figure 11a has illustrated the fabrication of novel MOFs meso-porous nFe_3_O_4_@MIL-88A(Fe)/*3*-aminopropyltrimethoxysilane (APTMS) nanocomposite, which exhibited excellent selective adsorption performance for heavy metal ions [184]. The maximum adsorption capacity for Cd^2+^, Pb^2+^ and Cr^2+^ achieved to 693.0, 536.22 and 1092.22 mg/g, respectively. It was worth noting that this nFe_3_O_4_@MIL-88A(Fe)/APTMS nanocomposite was easily regenerated and the adsorptive removal values were decreased by only 3% after five consecutive recycling processes. Safari et al., have developed a MMOFs composite consisting of phenylthiosemicarbazide MNPs (Fe_3_O_4_@PTSC) and MIL-101(Cr), and further utilized in MSPE to effectively isolate and simultaneously determine the heavy metals including Cd^2+^, Pb^2+^ and Ni^2+^ in agricultural and seafood samples [185]. By combining with flame atomic absorption spectrometry (FAAS), the developed MSPE method obtained the lower LODs (0.07–0.5 mg/kg), lower quantification limits (0.2–2.0 mg/kg), wide linear ranges (0.25–250 mg/kg) and good extraction recoveries (97.5–99.0%). Figure 11b illustrated a DNA fluorescence assay comprising a MMOFs functionalized with fluorescein amidite (FAM)-labeled ssDNA for Hg^2+^ detection [186]. The fluorescence intensity showed a drastic decrease upon the presence of Hg^2+^. The methods exhibited good sensitivity (LOD: 8 nM) and selectivity for Hg^2+^ over other co-existing metal ions. These merits made it an ideal platform for Hg^2+^ sensing applications. A novel MMOFs from dithizone-modified Fe_3_O_4_ nanoparticles and a copper-(benzene-1,3,5-tricarboxylate) MOF were successfully prepared and applied to the rapid extraction of trace quantities of heavy metal ions in fish, sediment, soil, and water samples [187]. By combined with FAAS, the LOD for Cd^2+^, Zn^2+^, Ni^2+^, and Pb^2+^ achieved to 0.12, 0.39, 0.98, and 1.2 ng/mL, respectively. Li et al., prepared Fe_3_O_4_@MOF@covalent organic framework (COF) by solvothermal methods for selectively separation and preconcentration of Cu^2+^ for the first time [188]. With a high density of nitrogen- and oxygen-containing functional groups on the surface, the Fe_3_O_4_@MOF@COF composite could efficiently extract and preconcentrate of Cu^2+^ ions in aqueous solutions, with a maximum adsorption capacity of 37.29 mg/g and LOD of 37.6 nm (*n* = 11) for Cu^2+^.

## 4. Conclusions

MMOFs have a unique structure and good adsorption performance and are easy to separate, which have become high-efficiency adsorbents widely used in the pretreatment process of food matrices and play an important role in the detection of trace contaminants in foods. (Table 2) However, the existing MMOFs still have the disadvantages of few kinds and poor stability, which cannot meet the different application requirements. The difficulties in large-scale preparation caused by relatively complicated synthesis process and the large difference between batches are also the factors restricting its further development.

Future development direction: (1) Green and efficient synthesis of MMOFs for food matrices pretreatment is very necessary. The exploration on the synthesis and application strategies of new, green, environmentally friendly and efficient MMOFs has become a new research hotpot. (2) The development and application of broad-spectrum MMOFs materials that can enrich and separate a variety of contaminants can significantly improve the pretreatment efficiency of food matrices and increase the detection throughput, which has broad application prospects. (3) Improving the matching of MMOFs materials and analytical instruments, and developing integrated, automated, online sample pretreatment technologies and equipment are of great significance to the detection and control of contaminants in foods.

## Figures and Tables

**Table 1:** Comparison of different strategies for MMOFs preparation.

Methods	Advantages	Disadvantages
Hybrid preparation method	Simple; suitable for most MMOFs	Easy to fall off
In-situ growth of MOFs on MNPs	Simple; synthesis at room temperature	Direct nucleation and growth of MOFs in solution
In-situ growth of MNPs on MOFs	More complete MOF structure; good adsorption property; remarkable magnetic response	Complex preparation process
Self-sacrificial template method	Core-shell structures; easy to operate; synthesis under mild conditions	Need a suitable precursor
Emulsion template method	Avoid embedding MNPs in MOFs tunnels; obtaining macroporous polymers	Require large amount of inorganic solvent; complex preparation process
LbL self-assembly method	Core-shell structure; precisely control the thickness and properties of MMOFs; synthesis at room temperature	Long preparation time Lack of self-assembled MOFs ligands
Dry gel conversion method	Avoid MNPs embedding in MOFs tunnels; reduce the loss of organic solvents	Not easily controlled uniformity of MOF growth
Mechanical grinding method	Synthesis at room temperature	Limited types of MOFs ligands; uneven MMOFs performance

**Table 2:** Analysis of Various Contaminants in Foods Using MMOFs as SPE Sorbent Combined with Different Instruments.

Materials	Analyte	Instrument	Sample	Conditions (Sorbent Amount; Eluent)	LOD	Linear Range	RSD	Recovery	Ref
Fe_3_O_4_/ZIF-8/IL	Pyrethroids	GC-MS/MS	Tea infusions (2 g)	10 mg; 0.8 mL of acetonitrile	0.0065–0.1017 μg/L	0.5–50 μg/L	≤9.70%	81.5–98.1%	[51]
Fe_3_O_4_@TGA@TMU-6	Phosalone Chlorpyrifos Profenofos	HPLC-UV	Rice (20 g)	2 mg; 0.1 mL of 1-butanol	0.15–0.87 µg/L	7.5–75 μg/L 10–100 μg/L 10–150 μg/L	4.8–7.3%	88–107.2%	[137]
MNPCs-Zn/Co-MOF	OPPs	GC-FPD	Fruit (1 g)	10 mg; 0.2 mL of acetone/ethyl acetate (1:1, *v*/*v*)	0.018–0.045 μg/L	0.1-100 μg/L	3.5–9.7%	84–116%	[138]
Nd_2_Fe_14_B-UIO-66, (Zr)-NH_2_	Sulfonylurea	HPLC	Water (5 mL)	5 mg; 0.2 mL of methanol-acetic acid (9:1, *v*/*v*)	0.04–0.84 μg/L	10–700 μg/L	≤13.8%	68.8–98.1%	[139]
Fe_3_O_4_@UiO-66-NH_2_@MON	Aflatoxins	HPLC	Corn, rice, millet (5 g)	10 mg; 6 mL of acetonitrile	1 μg/L	0.15–0.87 μg/L	–	87.3–101.8%	[154]
MIL53(Al)-SiO_2_@Fe_3_O_4_	Aflatoxin B1	FTIR	Herbal teas (no indication)	100 mg; 2 mL of Me_2_CO/MeCN/CH_2_Cl_2_ (1:1:2, *v*/*v*)	0.5 ng/mL	0.5–150 ng/mL	<4.3%	70.7–96.5%	[155]
Fe_3_O_4_/g-C3N4/HKUST-1	Ochratoxin A	Fluorescent biosensor	Corn (no indication)	–	2.57 ng/mL	5.0–160.0 ng/mL	<2.5%	96.5–101.4%	[157]
Fe_3_O_4_@PDA@Cu-MOFs	Microcystin	MALDI-TOF-MS	Water (80 μL)	20 μg; 10 μL of NH_4_HCO_3_ (0.25 mol/L)	0.015 mg/L	0.05–4 mg/L	6.1–8.2%	98.67–106.15%	[160]
Fe_3_O_4_ SPs@ZIF-8/Zn^2+^	Domoic acid	LC-MS	Shellfish (5 g)	1.0 mg; 0.4 mL of aqueous histidine solution (3 mmol/L)	0.2 pg/mL	1.0–1000 pg/mL	≤3.4%	93.1–102.3%	[161]
magG@PDA@ZIF-8	Phthalates	HPLC	Beverages (1 mL)	20 mg; 1 mL of acetone	0.003–0.09 ng/mL	50–8000 ng/mL	<4.5%	91.5–104.7%	[167]
Fe_3_O_4_-NH_2_@MIL-101	Sudan dyes	HPLC	Tomato (4 g)	3 mg; 1 mL × 2 ethyl acetate	0.5–2.5 μg/kg	0.01–25 μg/mL	≤9.2%	69.6–92.6%	[169]
Fe_3_O_4_@ 1-phenylthiosemicarbazide/MIL-101(Cr)	Cd^2+^; Pb^2+^; Ni^2+^	FAAS	Shrimp, Cucumber, Tomato, Parsley (1 g)	13 mg; 2.2 mL of 0.85 mol/L HCl	0.07–0.5 mg/kg	0.07–0.5 mg/kg 0.2–2.0 mg/kg 0.25–250 mg/kg	4.5–7.3%	97.5–99.0%	[185]
Fe_3_O_4_-NH_2_@MIL-101(Fe)	Hg^2+^	Fluorescent biosensor	Water (no indication)	–	8 nm	2–20 nm	--	About 70%	[186]
M-MOF/β-CD	Triazole Fungicides	HPLC-MS/MS	Lettuce, tomato (10 g)	10 mg; 4 mL of acetone-sodium citrate buffer	0.25–1.0 mg/L	0.25–1.0 μg/L	1.5–7.3%	86.44–119.83%	[189]
Fe_3_O_4_@JUC-48	Sulfonamides	HPLC	Chicken, pork, shrimp (5 g)	25 mg; 0.8 mL of methanol	3.97–1000 ng/g	3.97–1000 ng/g	<4.5%	76.1–102.6%	[190]
